# DF-4 defibrillator downgrade to pacemaker, a novel method of device downgrade for implantable cardioverter-defibrillator patients

**DOI:** 10.1016/j.hrcr.2023.07.012

**Published:** 2023-08-15

**Authors:** Christopher Monkhouse, James Elliott, Jason Collinson, Ross Hunter, Pier Lambiase, Syed Ahsan, Philip Moore

**Affiliations:** Barts Heart Centre, London, United Kingdom

## Introduction

Downgrading implantable cardioverter-defibrillators (ICD) is becoming a common clinical scenario in an aging population with increased comorbidities and more widespread use of do-not-resuscitate orders. Therefore, more patients have had ICD therapies deactivated. However, when devices require replacement, it is unnecessary to implant a larger, more expensive defibrillator to keep therapies turned off. We describe a potential solution for patients using current technology.

## Case histories

**Figure 1:**
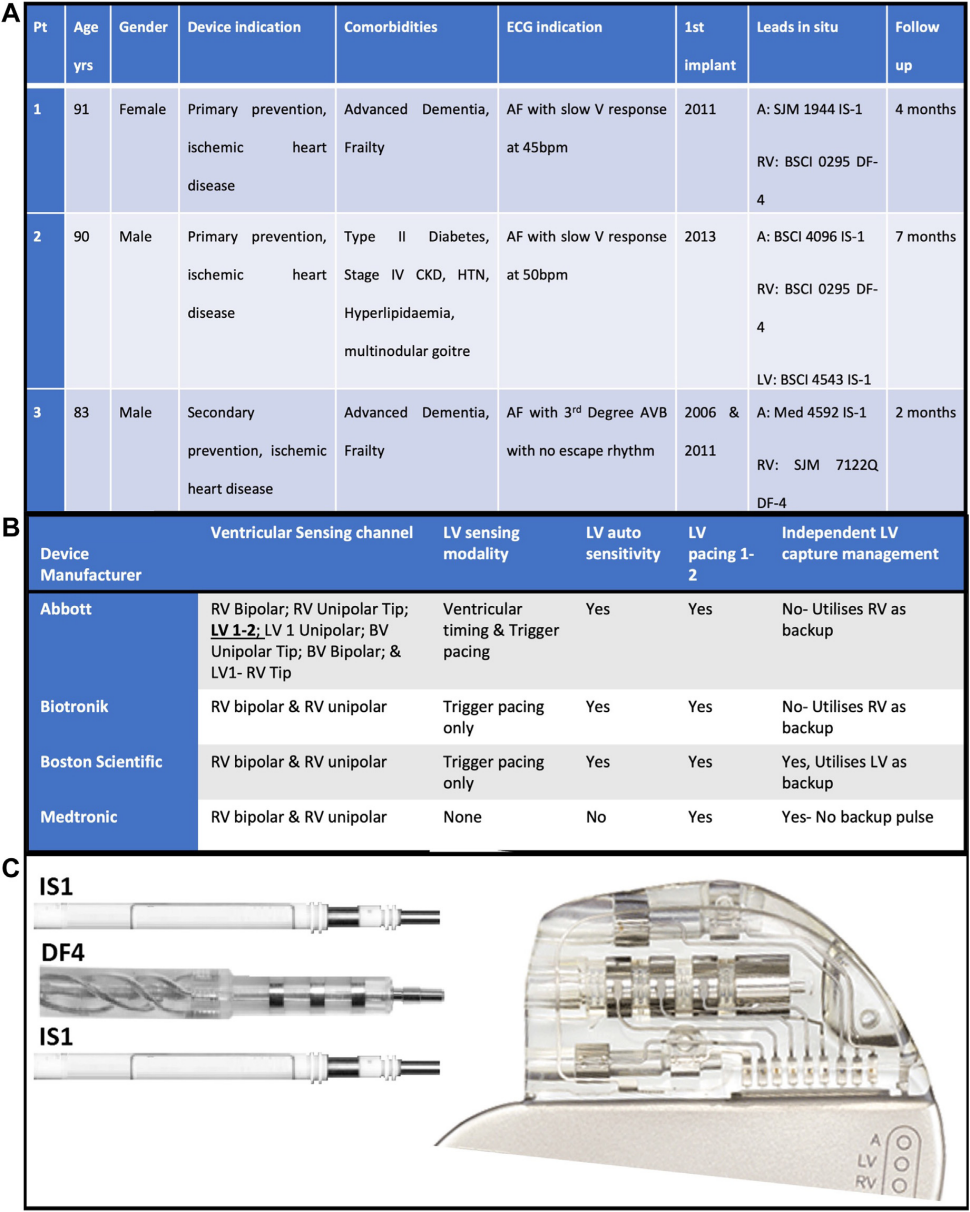
**A:** Patient demographics. **B:** Programming features of IS-4 cardiac resynchronization pacemaker (CRT-P) devices. **C:** Schematic of leads and CRT-P generator for patient 2; for other patients the second IS-1 port was plugged. A = atrial; AF = atrial fibrillation; AVB = atrio-ventricular block; BSCI = Boston Scientific; CKD = chronic kidney disease; ECG = electrocardiogram; HTN = hypertension; LV = left ventricular; Med = medtronic; RV = right ventricular; SJM = St Jude Medical; V = ventricular.

Three patients with ICDs and DF-4 right ventricular (RV) leads were referred for generator change procedures owing to expected battery depletion. All patients had do-not-resuscitate orders in place and were at various stages of palliative care owing to noncardiac comorbidities. Demographics are displayed in Figure 1A. All patients had management options discussed in a consultant-led, specialist device clinic with relatives or legal power of attorney. All patients received remote monitoring for their uneventful follow-up, prior to dying of non-pacing-related causes.

## Methods

Downgrading a DF-4 ICD lead requires a specific manufacturer device owing to programming requirements. An Abbott (Chicago, IL) cardiac resynchronization pacemaker (CRT-P) device with an international standard-4 (IS-4) left ventricular (LV) port is used because of the ability to program the LV channel as the ventricular sensing channel and, importantly, timing cycle calculations. We summarize the programmability of CRT-P generators in Figure 1B.

Thus, when downgrading, the DF-4 RV lead is placed in the IS-4 LV port. The IS-4 LV port is compatible with a DF-4 pin, as step-down components of the DF-4 lead are not obstructed. The mechanics are commonly misunderstood: the IS-4 pin will not fit in a DF-4 port, but a DF-4 pin will fit in an IS-4 port (Figure 1C). Significantly, the International Organization of Standardization documentation for quadripolar connectors shows that the dimensions for both IS-4 and DF-4 have the same screw contact, ensuring tight engagement.^1^

The ventricular sensing and pacing should then be programmed to LV 1–2, which corresponds to the RV tip and ring electrodes. CapConfirm™ should be disabled, as the backup pulse is delivered through the RV port. If the patient has downgraded from a CRT device, the CRT offset timing needs to be reversed (eg, LV-20 ms would be RV-20 ms).

The lead configurations should be highlighted on the device using the notes function and the importance of not changing the sensing channel documented.

## Discussion

Downgrading a DF-4 lead to IS-4 has previously been reported in a single case report by Giedrimas and colleagues^2^ in 2018 using a Boston Scientific (Marlborough, MA) CRT-P generator. Importantly, this required the LV IS-1 pin to be placed in the RV IS-1 port to use ventricular sensing through the RV port. We provide an alternative for patients who do not have an LV lead, making our method an option for patients with DF-4 ICDs and patients who have an LV IS-1 connector CRT defibrillator. Unfortunately, there is no solution for patients with an IS-4 LV lead and a DF-4 RV lead.

Reducing the risk from lead intervention and procedural complexity is vital in complex patients who have limited life expectancy, and financial implications are important considerations. A CRT-P device costs approximately two-thirds of an ICD.^3^^,^^4^

## Conclusion

We demonstrate the successful downgrade of 3 DF-4 ICD patients using an Abbott CRT-P generator with the DF-4 RV lead placed in the LV IS-4 port to ensure sensing and pacing functions. Furthermore, we described why this device is preferable in this setting.
